# Acute Biliary Pancreatitis During Pregnancy: Time to Step Up Our Efforts!

**DOI:** 10.1002/ueg2.70111

**Published:** 2025-10-09

**Authors:** Robert C. Verdonk

**Affiliations:** ^1^ St. Antonius Hospital Nieuwegein Utrecht the Netherlands

Pregnancy is associated with an increased risk of biliary symptoms. Due to hormonal changes the motility of the gallbladder decreases, the composition of the bile changes and becomes more lithogenic. The rising prevalence of obesity and increasing age at the time of pregnancy add to the increase in biliary problems [1]. One of the most feared complications of gallstones during pregnancy is acute biliary pancreatitis (ABP). The combination of pregnancy and acute pancreatitis leads to substantial concern in both gastroenterology and obstetric care givers since especially severe acute pancreatitis increases the risk of preterm delivery and perinatal mortality.

In this issue of the UEG Journal, Tarjan et al. describe the results of the BORN‐study [2]. The authors describe their multicenter, international cohort study, looking at management and outcome of patients admitted with ABP during pregnancy. A specific focus of this paper is the use of Endoscopic Retrograde Cholangiopancreaticography (ERCP) and cholecystectomy, and the impact of these treatments on the outcome of pregnancy. In total 101 patients were included from 19 centers, with a median gestational age of 25 weeks. The majority of patients had mild pancreatitis (86%). As a treatment, 29 cholecystectomies were performed, and ERCP was performed in 22 patients. A major finding in the present cohort is the impact of cholecystectomy on recurrent gallstone related complications: 0% in the cholecystectomy group versus 24% in the group that did not undergo surgery. Both cholecystectomy and ERCP appeared safe with low numbers of fetal and maternal adverse events and no impact of cholecystectomy on fetal loss.

The authors should be complimented for performing this study: the subject is of increasing importance, patients from multiple counties were included, and great effort was put in obtaining source data.

Several aspects of this stud are worth a closer look.

First, it shows that we have to step up our efforts. A large body of evidence including randomized controlled trials support same admission cholecystectomy for ABP [3]. In this study a cholecystectomy was performed in only 29 of 101 patients. A similar pattern is described in previous studies [1, 4] with cholecystectomy rates of approximately 50%. Additionally, present [1] and previous [4] studies show that if one refrains from adequate management including cholecystectomy, the rate of re‐admissions and subsequent biliary complications is very high. So despite our best intentions as gastroenterologist and surgeons, there appears to be a reluctance to perform cholecystectomy in pregnant patients, leading to the exact opposite of our intention: more complications. It is therefore of importance that recent international guidelines [5] on the management of acute pancreatitis explicitly states that ABP during pregnancy should be managed in the same manner as in non‐pregnant patients: with early cholecystectomy and ERCP if indicated. The work by Tarjan et al. supports these recommendations.

Another issue discussed in the present paper is the use of ERCP during pregnancy. It is fairly difficult to understand from the paper in how many cases of proven choledocholithiasis a conservative approach was chosen. Previous studies show that pregnant patients are less likely to undergo ERCP than non‐pregnant patients with ABP [2, 4]. One of the important takes from the present study and previous studies and guidelines is that ERCP during pregnancy is safe. Outcomes are generally excellent, radiation exposure to the fetus is low and well below the threshold of fetal damage [6, 7]. It goes without saying that the indications for ERCP during pregnancy are similar to those outside pregnancy: acute cholangitis and persistent choledocholithiasis.

A major consideration when contemplating ERCP during pregnancy is to confirm there is persistent choledocholithiasis, even if previous imaging has shown a bile duct stone. Many stones pass spontaneously, and even in suspected acute cholangitis there is a substantial rate of stone‐negative ERCP [8]. In the Tarjan study, gallstones were conformed in only 71% of patients undergoing ERCP. I would strongly advice to do endoscopic ultrasonography prior to introduction the duodenoscope in all pregnant patients to confirm there is a solid indication to proceed.

Several potential sources of bias should be considered when interpreting the present study, most also mentioned by the authors. This is a retrospective study. The time‐span of inclusion was from 2011 onward, so it could be that we are looking at a different era of pancreatitis care. Importantly, only 101 patients were include from 19 centers in a 13 years' timeframe leading to less than 1 patient per center per year, which should make us aware of the risk of missing cases. Finally, the low number of cases undergoing ERCP and/or cholecystectomy should make us cautious to draw too firm conclusions.

The study by Tarjan et al. in this UEG Journal highlight the potential for improvement when taking care of pregnant patients with acute biliary pancreatitis. Pregnancy should not withhold us to provide optimal care, but should encourage us to do so, supported by evidence that it is safe and effective.

## Conflicts of Interest

The author declares no conflicts of interest.

## Data Availability

The author has nothing to report.
